# Regarding EpCAM-targeted near-infrared photoimmunotherapy (NIR-PIT) for the treatment of breast cancer

**DOI:** 10.1080/07853890.2025.2560608

**Published:** 2025-09-18

**Authors:** Wei Mao, Yong Chen, Zegang Liu

**Affiliations:** ^a^ Chuxiong Yi Autonomous Prefecture Maternal and Child Health Care Hospital, Chuxiong, China; ^b^ 920th Hospital of People’s Liberation Army Joint Logistic Support Force, Kunming, China

To the Editor

We perused with keen interest the recent article entitled ‘EpCAM-targeted near-infrared photoimmunotherapy (NIR-PIT) for the treatment of breast cancer’ [1] published in your esteemed journal. The authors deserve commendation for their innovative methodology in extending the therapeutic potential of NIR-PIT beyond EGFR-targeted therapies. Their research meticulously showcases the efficacy of EpCAM-targeted NIR-PIT in both human and murine breast cancer models, especially when combined with immune checkpoint inhibitors (ICIs). The induction of robust CD8+ T cell activation and long-term anticancer immunity, as evidenced by a 50% complete remission rate in the combination therapy group, underscores the translational promise of this strategic approach.

Notwithstanding these strengths, we contend that several aspects merit further deliberation to inform future research and clinical applications. Firstly, although the study underscores the selective targeting of EpCAM-expressing cancer cells, the observed decline in NK cells, macrophages, and MDSCs – presumably attributable to Fc receptor binding – gives rise to concerns regarding potential off-target effects on immune cell populations that are crucial for antitumor responses. Future investigations would stand to gain from more in-depth immunophenotyping to elucidate the equilibrium between targeted cytotoxicity and inadvertent immune modulation.

Secondly, the selection of Edrecolomab as the anti-EpCAM antibody, although a pragmatic choice considering its clinical safety profile, might potentially restrict therapeutic efficacy owing to its relatively low affinity. The authors’ cautious remarks regarding high-affinity clones such as ING-1 (for instance, sensitivity to ambient light) are well-founded. However, investigating intermediate-affinity antibodies (e.g. adecatumumab) could potentially optimize the balance between efficacy and safety. Moreover [2,3], although the study’s reliance on subcutaneous tumor models provides valuable insights, it may not comprehensively replicate the heterogeneity and immune microenvironment characteristic of orthotopic or metastatic breast cancer.

Ultimately, the clinical applicability of EpCAM-NIR-PIT is contingent upon the accurate delivery of light to prevent collateral damage to EpCAM-positive normal tissues, such as the skin and colon. The authors’ proposal of endoscopic NIR light application for visceral tumors is perceptive. However, technical hurdles regarding dose standardization and real-time monitoring still need to be resolved.

Within the framework of promoting personalized cancer therapy, this research highlights the potential of EpCAM–NIR–PIT as a multimodal platform. We advocate for further in-depth investigations into its integration with other immune-modulating agents (such as cytokines and bispecific antibodies) and its assessment in metastatic scenarios. The authors’ work lays the foundation for a new era of antigen-targeted phototherapies. We eagerly anticipate witnessing its progress towards clinical trials.

## Data Availability

Data sharing is not applicable to this article as no data were created or analysed in this study.

## Author’s information

Wei Mao, Yong Chen

Chuxiong Yi Autonomous Prefecture Maternal and Child Health Care Hospital, Chuxiong, China

Zegang Liu

Chinese People’s Liberation Army Joint Support Force No. 920 Hospital, Kunming, China

likerong64@126.com

## References

[1] Furusawa A, Takao S, Suzuki M, et al. EpCAM-targeted near-infrared photoimmunotherapy (NIR-PIT) for the treatment of breast cancer. Ann Med. 2025;57(1):2540599. doi: 10.1080/07853890.2025.2540599. 40792441 PMC12344674

[2] Münz M, Murr A, Kvesic M, et al. Side-by-side analysis of five clinically tested anti-EpCAM monoclonal antibodies. Cancer Cell Int. 2010;10(1):44. doi: 10.1186/1475-2867-10-44. 21044305 PMC2989956

[3] Eyvazi S, Farajnia S, Dastmalchi S, et al. Antibody based EpCAM targeted therapy of cancer, review and update. Curr Cancer Drug Targets. 2018;18(9):857–868. doi: 10.2174/1568009618666180102102311. 29295696

